# The Effect of Dopaminergic Therapy in Parkinson’s Disease: A Graph Theory Analysis

**DOI:** 10.3390/brainsci15040370

**Published:** 2025-04-02

**Authors:** Karthik Siva, Palanisamy Ponnusamy, Vishal Chavda, Nicola Montemurro

**Affiliations:** 1 Department of Electronics and Communication Engineering, National Institute of Technology, Tiruchirappalli 620015, India; shivakarthik.grylls@gmail.com; 2 Department of Medicine and Critical Care, Multispeciality, Trauma and ICCU Centre, Sardar Hospital, Ahmedabad 380024, India; chavdavishal2@gmail.com; 3 Department of Neurosurgery, Azienda Ospedaliero Universitaria Pisana, 56100 Pisa, Italy; 4 EndoCAS Interdipartimental Center, University of Pisa, 56100 Pisa, Italy

**Keywords:** Parkinson’s disease, dopaminergic therapy, clustering coefficient, participation coefficient, small-worldness, graph theory, normalization effect

## Abstract

**Background**: Dopaminergic therapy (DT) is the gold standard pharmacological treatment for Parkinson’s disease (PD). Currently, understanding the neuromodulation effect in the brain of PD after DT is important for doctors to optimize doses and identify the adverse effects of medication. The objective of this study is to investigate the brain connectivity alteration with and without DT in PD using resting-state EEG. **Methods**: Graph theory (GT) is an efficient technique for analyzing brain connectivity alteration in healthy and patient groups. We applied GT analyses on three groups, namely healthy control (HC), Parkinson with medication OFF (PD-OFF), and Parkinson with medication ON (PD-ON). **Results**: Using the clustering coefficient (CC), participation coefficient (PC), and small-worldness (SW) properties of GT, we showed that PD-ON patients’ brain connectivity normalized towards healthy group brain connectivity due to DT. This normalization effect appeared in the brain connectivity of all EEG frequency bands, such as theta, alpha, beta-1, beta-2, and gamma except the delta band. We also analyzed region-wise brain connectivity between 10 regions of interest (ROIs) (right and left frontal, right and left temporal, right and left parietal, right and left occipital, upper and lower midline regions) at the scalp level and compared across conditions. During PD-ON, we observed a significant decrease in alpha band connectivity between right frontal and left parietal (*p*-value 0.0432) and right frontal and left occipital (*p*-value 0.008) as well as right frontal and right temporal (*p*-value 0.041). **Conclusion**: These findings offer new insights into how dopaminergic therapy modulates brain connectivity across frequency bands and highlight the continuous elevation of both the segregation and small-worldness of the delta band even after medication as a potential biomarker for adverse effects due to medication. Additionally, reduced frontal alpha band connectivity is associated with cognitive impairment and levodopa-induced dyskinesia, highlighting its potential role in Parkinson’s disease progression. This study underscores the need for personalized treatments that address both motor and non-motor symptoms in PD patients.

## 1. Introduction

Parkinson’s disease (PD) is a neurodegenerative disease characterized by motor symptoms, such as tremors, rigidity, and bradykinesia, as well as non-motor symptoms, including cognitive decline, mood disturbances, hyposmia, and sleep disorders [1,2]. These deficits make it challenging for patients with Parkinson’s disease to perform daily tasks effectively. Emerging evidence suggests that endocrine factors and neuropeptide dysregulation, such as orexin abnormalities, contribute to Parkinson’s disease pathophysiology. Orexin, a hypothalamic neuropeptide involved in wakefulness, autonomic regulation, and metabolic processes, has been linked to non-motor symptoms, including sleep dysfunction in PD [3]. Among non-pharmacological treatment approaches, deep brain stimulation (DBS) aimed at the subthalamic nucleus (STN) has demonstrated notable benefits in motor function [4]. However, only a small fraction of patients are considered suitable candidates for this procedure, and it is associated with significant adverse effects. Conversely, transcranial direct current stimulation (tDCS) targeting the dorsolateral prefrontal cortex has provided transient improvements in motor symptoms in almost half of PD patients. Dopamine, a crucial neurotransmitter involved in several high-order brain functions, plays a significant role in neural pathways originating from the brainstem [5]. These dopaminergic pathways, including the nigrostriatal, mesolimbic, and mesocortical systems, are integral in managing motor control, cognition, and emotional regulation [6,7]. Furthermore, dopamine is believed to influence brain network connectivity through its projection routes. It involves dopaminergic signals from the striatum that activate regions such as the dorsolateral prefrontal cortex, the supplementary motor area, and the cingulate gyrus. Various dopaminergic medications have been used for treating PD, including levodopa/carbidopa, Amantadine, entacapone, pramipexole, rasagiline, ropinirole, and Selegiline [8]. These medications have shown improvements in motor function in PD patients. Treatment strategies are tailored based on disease stage, symptom profile, and patient characteristics. In the early stage, dopamine agonists such as pramipexole and ropinirole may be used to delay levodopa introduction, whereas monoamine oxidase-B (MAO-B) inhibitors like rasagiline provide mild symptomatic relief. As the disease progresses, levodopa, often combined with a decarboxylase inhibitor (carbidopa), becomes the mainstay of treatment, supplemented by catechol-O-methyltransferase (COMT) inhibitors (e.g., entacapone) to prolong its efficacy [9]. For patients with advanced PD experiencing motor fluctuations, continuous dopaminergic stimulation via duodenal levodopa/carbidopa gel infusion or subcutaneous apomorphine infusion may be beneficial [10]. Additionally, deep brain stimulation (DBS) is an effective option for refractory motor symptoms [11]. Importantly, while levodopa effectively improves motor symptoms, its impact on non-motor symptoms such as depression remains complex [12]. Limitations in study designs, such as poor methodological rigor, have hindered the ability to draw definitive conclusions about medication effect. The absence of control groups with medication complicates the task of differentiating the drug’s effects from natural disease progression [13].

Dopaminergic therapy remains a potentially effective and safe treatment for PD. Nonetheless, a rigorous and standardized evaluation of its efficacy, alongside a clearer understanding of how it affects PD patients’ brain networks and identifies responders, is still needed [14]. While EEG provides high temporal resolution and practical advantages for clinical monitoring, alternative neuroimaging techniques like functional MRI (fMRI) and magnetoencephalography (MEG) offer complementary insights. fMRI captures hemodynamic changes with high spatial resolution but lacks the millisecond-scale precision needed to track rapid neural dynamics in PD. MEG combines strong spatial and temporal resolution but is limited by cost, restricted availability, and sensitivity to movement artifacts—a significant constraint for PD patients with motor symptoms. In contrast, EEG’s portability, affordability, and tolerance to movement make it uniquely suited for longitudinal and bedside assessments of dopaminergic therapy effects in PD, despite its lower spatial resolution. Advances in technology now enable resting-state EEG recordings, providing a platform to explore neuronal functions and brain connectivity [15]. While spectral power analysis is a well-established method for examining EEG data and has extensive literature supporting its connection to various cognitive processes and states, it alone may not fully explain the recovery process in response to medication [16].

Given that the normalization effects of dopaminergic therapy involve coordinated changes across the entire brain network, a more comprehensive approach that considers the interactions between neuronal populations is necessary to understand the effects of medication better. This study originated from the hypothesis that graph theory, an efficient mathematical technique, is used to demonstrate the brain connectivity normalization effect after dopaminergic therapy in PD.

Extensive research into brain function has utilized graph theory to map brain connectivity [17], a modern approach that is increasingly applied to studies of pharmacological neuromodulation. There is increasing attention on understanding the brain’s network topology, as it offers mechanistic insights that could explain distinct patterns of altered connectivity, going beyond simple synchronization between brain regions [18]. Brain activity and neural synchrony typically exhibit unique topological arrangements, with segregated modules characterized by dense short-range connections that serve distinct functions. These modules are also interconnected by long-range connections that facilitate integration both within and between them [19,20].

The brain’s topology exhibits small-world network characteristics during different states by integrating distinct functional modules through network hubs, which facilitate efficient communication. Additionally, a balance between segregation and integration is observed during higher cognitive functions [21], while this balance is disturbed in conditions such as Parkinson’s disease due to pathological factors. Therefore, beyond brain connectivity, the network’s topological structure, often assessed through graph theory methods, can offer additional insights into topological alterations. These insights may complement the whole-brain connectivity changes associated with the neural mechanisms’ underlying medication effects.

Building on our previous findings [22] in fMRI data, where the clustering coefficient (CC) and small-worldness (SW) emerged as the top-ranked features for classification, we hypothesize that these graph metrics—along with the participation coefficient (PC)—will similarly enhance brain connectivity accuracy in EEG analysis, regardless of modality. Their demonstrated sensitivity in distinguishing groups suggests their robustness as key predictors in classification algorithms. Among various rehabilitation techniques, our primary focus is on dopamine therapy and evaluating its effects on brain topology. We used resting-state EEG, which reflects task-independent brain states, to offer insights into medication effect on Parkinson’s disease. Using power spectral and graph theory techniques, we assessed the impact of dopamine therapy on brain topology in Parkinson’s patients [23,24]. Our study involves three groups: (a) healthy controls, (b) Parkinson’s patients with medication OFF, and (c) Parkinson’s patients with medication ON, with fifteen participants in each group. We evaluated them using resting-state EEG before and after dopamine therapy. Resting-state functional connectivity provides extensive information about the brain’s functional networks, potentially overcoming the limitations of task-based methods [25].

Comparisons between the treatment and non-treatment groups will help determine the effects of dopamine therapy on brain function in Parkinson’s patients. Understanding these changes in brain connectivity could contribute to uncovering the underlying mechanisms of Parkinson’s disease and developing improved therapeutic strategies.

## 2. Materials and Methods

### 2.1. Resting-State EEG Data

**Figure 1:**
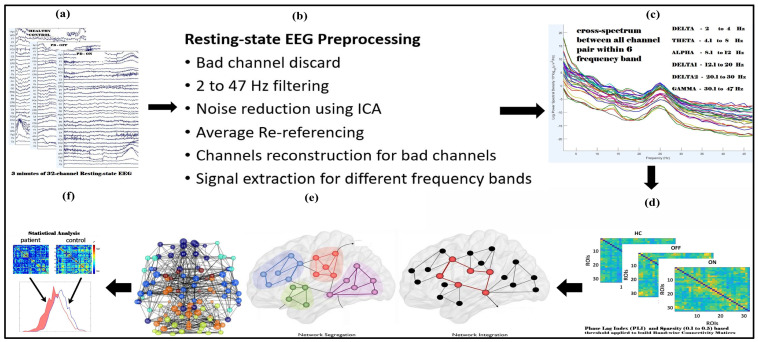
Flowchart illustrating the complete steps for resting-state EEG (rsEEG) analysis used performing graph theory analysis in the human brain. The steps include rsEEG data collection (**a**), preprocessing (**b**), frequency band-wise separation (**c**), constructing PLI connectivity matrices for each frequency band (**d**), and thresholding and binarizing these matrices, performing graph theory analysis (**e**), and statistical analysis (**f**).

The resting-state EEG data analyzed in this study were downloaded from an OpenNeuro website, OpenNeuro Accession Number ds002778 [26]. They include approximately 3 min of resting-state EEG recordings from 15 healthy controls (9 females, aged 63.5 ± 9.6 years) and 15 patients with Parkinson’s disease (8 females, mean age = 63.2 ± 8.2 years, 7 males, ages 62.29 ± 9.69 years), all of whom had mild to moderate disease with an average disease duration of 4.5 ± 3.5 years. Participants’ ages did not differ between PD and control groups (*p* = 0.94), reducing the likelihood that observed network changes were driven by aging rather than PD pathology. Dopaminergic therapy for the management of Parkinson’s disease includes a variety of medications, such as Amantadine, entacapone, and carbidopa/levodopa, in both regular and sustained-release formulations. Additionally, dopamine agonists such as ropinirole (both regular and extended-release) and pramipexole are utilized, along with monoamine oxidase-B (MAO-B) inhibitors like rasagiline and Selegiline. It is important to note that dosages of these medications can vary significantly among patients. In this study, 13 out of 15 patients commonly received carbidopa/levodopa at a dosage of 25/100 mg and rasagiline at 1 mg. EEG data were collected during both OFF and ON dopaminergic medication states. All participants were right-handed, and written informed consent was obtained in accordance with the guidelines of the University of California, San Diego Institutional Review Board, and the Declaration of Helsinki. Further details regarding the clinical characteristics of all patients can be found in George et al. 2013 [27]. EEG recordings for Parkinson’s disease patients were conducted on two separate days, corresponding to ON and OFF medication sessions, with the order of sessions counterbalanced among participants. For the OFF session, patients discontinued their medication at least 12 h before the EEG recording. During the ON session, patients took their medication as prescribed. The patients were closely matched with healthy controls based on age, gender, and handedness. A 32-channel EEG cap connected to the BioSemi ActiveTwo system was used for data acquisition at a sampling rate of 512 Hz, with additional electrodes placed over the left and right mastoids for referencing. Participants were instructed to sit comfortably and focus on a cross displayed on the screen during the recording, which lasted for at least 3 min. In addition, patients completed several clinical assessments, Mini-Mental Status Exam, North American Adult Reading Test, Beck Depression Inventory (BDI), Unified Parkinson’s Disease Rating Scale; detailed information regarding the clinical condition of patients and healthy control subjects is available in Table 1 and Table 2 of the original publication by George et al. 2013 [27]. The BDI of Patients Off and On versus controls was t27 = 3.05, *p* < 0.01 and t30 = 4.25, *p* < 0.005, respectively. Figure 1 illustrates the complete pipeline for resting-state EEG (rsEEG) analysis, including data collection, preprocessing, frequency band separation, PLI connectivity matrix construction, graph theory analysis, and statistical evaluation.

### 2.2. Preprocessing

The EEG data were preprocessed using EEGLAB version 14.1.2 [28], TAPEEG toolboxes [29], and custom scripts in MATLAB R2024a (The MathWorks, Portola Valley, CA, USA). Initially, the EEG signals were re-referenced to the scalp average, followed by applying a high-pass filter at 0.5 Hz to eliminate low-frequency drifts. The Clean-line plugin was employed to remove 50 Hz line noise. Channels with poor signal quality were identified and excluded through visual inspection, and their data were replaced using spline interpolation. Independent component analysis (ICA) with the infomax algorithm was implemented in EEGLAB to identify and remove artefacts related to cardiac activity, eye movements, blinks, and muscle noise. The artefact components were discarded, and the remaining signals were reconstructed by back projecting the non-artifactual components. In total, 3 min of EEG data were used for post-processing analysis. Power spectral densities for recording were calculated across six frequency bands of interest (delta: 2–4 Hz; theta: 4.1–8 Hz; alpha: 8.1–12 Hz; beta-1: 12.1–20 Hz; beta-2: 20.1–30 Hz; and gamma: 30.1–47 Hz) using a Hanning-windowed fast Fourier transform.

### 2.3. Connectivity Analysis

For each frequency band, connectivity between electrodes was calculated using the phase lag index. The phase relationships between EEG signals offer valuable insights into the functional connectivity between brain regions. The phase lag index (PLI) is commonly used to assess the asymmetry in phase differences between two signals, with the added advantage of being less influenced by common sources such as volume conduction. The mathematical limits on the differences in instantaneous phases, derived using the Hilbert phase transform, reveal dynamic entrainment between the time series. The phase synchronization between the two time series is represented by the following:

(1) $$\left|\emptyset_{n,m}\right|=\left|\emptyset_{1}-\emptyset_{2}\right|<const$$

where $\emptyset_{1}$ and $\emptyset_{2}$ are the instantaneous phases of the two time series derived from the Hilbert transform [27] of the two time series:

(2) $$z\left(t\right)=x\left(t\right)+ix\left(t\right)=A(t)e^{i\emptyset t}$$

PLI-based connectivity networks are constructed by examining a consistent phase lag between the instantaneous phases of two electrodes. While synchronization from common sources typically occurs at zero lag, the PLI captures a more biologically realistic, time-dependent synchrony that arises from underlying neuronal interactions [30].

The PLI excludes phase distributions that are concentrated around 0 mod π.

(3) $$PLI=\left|<sign(∆\emptyset_{rel}\left(t\right))>\right|=\left|\frac{1}{N\;}\sum_{n=1}^{N}sign(∆\emptyset_{rel}(t_{n}))\right|$$

### 2.4. Graph Theory Measure

We analyzed brain topological organization using a graph theory approach. We analyzed popular graph measures such as network segregation (clustering coefficient), network integration (participation coefficient), and small-worldness.

The clustering coefficient quantifies local connectivity by measuring how well a node’s neighboring regions are interconnected. Neurophysiologically, this reflects the brain’s ability to form functionally specialized modules that support specific cognitive and sensory processes. A high clustering coefficient suggests strong local communication, which is essential for efficient information processing within specialized regions, such as the motor and sensory cortices. The participation coefficient assesses how well a node connects to different functional modules within the network, capturing its role in integrating information across brain regions. From a neurophysiological perspective, high participation coefficients are indicative of hub regions that facilitate global information transfer, such as the prefrontal cortex, which integrates sensory, motor, and cognitive functions. A disruption in this measure has been linked to impaired cognitive flexibility and executive dysfunction. The average path length measures global connectivity by determining the shortest number of steps needed to transfer information between any two brain regions. Neurophysiologically, shorter path lengths indicate more efficient information flow, supporting rapid communication across distant brain areas. A prolonged path length is often associated with disrupted connectivity, as observed in neurodegenerative conditions where interregional communication becomes inefficient. Finally, small-worldness describes an optimal balance between local specialization and global integration, mirroring the brain’s efficient network organization. A small-world topology enables both rapid long-range communication and functionally distinct processing hubs, a characteristic of healthy brain function. Alterations in small-world properties have been reported in neuropsychiatric and neurodegenerative diseases, reflecting disruptions in the brain’s ability to efficiently coordinate complex cognitive and motor functions.

Network Topology Analysis: Graph measures were computed using phase lag index (PLI)-based connectivity matrices. The brain networks were represented as an N × N (i.e., 32 × 32) undirected binary graph, denoted as G(N,E), where G signifies the subset of non-zero elements. In this representation, E corresponds to the edges, which are coefficients obtained from intermodal correlations, while N represents the nodes, defined as the regions of interest (ROIs) or electrodes involved in connectivity. This study employs an undirected binary network framework to analyze network characteristics, facilitating a direct comparison of nodal properties across different participants and groups. A sparsity thresholding approach was applied to ensure uniform network edge density across all participants, retaining only the strongest connections that exceeded a predetermined threshold. To prevent excessive fragmentation at lower densities, sparsity values were systematically varied from 10% (S = 0.1) to 50% (S = 0.5) in increments of 0.05.

Clustering coefficient: The network segregation is quantified using the clustering coefficient (CC) by computing the triangles of nodes and those that are found in the neighborhood of the nodes. The CC primarily measures the network’s local interconnectivity and the level up to which the networks can be segregated effectively. For calculating the CC, the technique as followed by Watts et al., 1998 [31] is employed, which defines that the weights between the ‘i_th_’ node and the rest of the ‘j’ nodes must be symmetrically arranged. The ratio of the count of prevailing interconnections to the count of all possible interconnections among the neighborhood nodes is indicated by ‘C_i_’ for that corresponding node. The CC refers to the normalized value of the absolute CCs of all the nodes present in the topological system. This clustering coefficient can be mathematically expressed as follows:

(4) $$CC=\frac{1}{n}\sum_{i\in N}C_{i}$$

where $C_{i}=\frac{2E_{i}}{Q_{i}(Q_{i}-1)}$, where N refers to the total count of nodes present in the network, E_i_ indicates the count of the existing links of the i_th_ node, where the degree of node i is denoted by Q_i_; this Q_i_ indicates the extent to which the node stays linked with the remaining nodes in the brain topology. A node with the highest Q_i_ measure has the highest number of links with the rest of the nodes.

Participation coefficient: The participation coefficient is a measure that reflects the diversity of connections a node has across different network modules. The Louvain algorithm was applied to identify these modules using a resolution parameter set to one. This algorithm optimizes the community structure by maximizing the number of connections within modules while minimizing those between them [32]. The participation coefficient was calculated for each node by assessing its connections within each module. The participation coefficient was calculated by the following:

(5) $$y_{i}=1-\sum_{m\in M}{\left(\frac{k_{i}(m)}{k_{i}}\right)}^{2}$$

where k_i_(m) represents the degree of connections between node i and nodes in that module m.

Small-worldness: An actual network is a small world when that network satisfies the two criteria mentioned as follows: $\gamma =\frac{C_{i}}{C_{Rand}}>1$ and $\lambda =\frac{L_{i}}{L_{Rand}}\approx 1$; Li and Ci are the path length and absolute clustering coefficient, respectively, where C_Rand_ and L_Rand_ are the average clustering coefficient and characteristic path length of the coordinated random networks [32]. A topology is known as an organization of a small world when the network satisfies the condition $\sigma =\frac{\gamma }{\lambda }>1$.

### 2.5. Statistics

We verified the assumptions of normality and homogeneity of variance. Shapiro–Wilk tests confirmed that all groups (HC, PD-OFF, PD-ON) were normally distributed for the clustering coefficient (CC; *p* ≥ 0.79), participation coefficient (PC; *p* ≥ 0.50), and small-worldness (SW; *p* ≥ 0.89). Levene’s test further confirmed the homogeneity of variance across groups (CC: *p* = 0.074; PC: *p* = 0.541; SW: *p* = 0.126). As assumptions were satisfied, Student’s t-tests were appropriately employed for group comparisons.

For the Beck Depression Inventory (BDI) scores, Shapiro–Wilk tests confirmed normality across all groups (HC: *p* = 0.209; PD-OFF: *p* = 0.554; PD-ON: *p* = 0.983). Levene’s test indicated homogeneity of variance (*p* = 0.221).

The graph theory measures were examined with the range of 0.1 (10%) to 0.5 (50%) sparsity thresholds in all nodes for each patient, and healthy control and two-sample two-tailed t-tests were used to calculate the group difference. Multiple comparisons were corrected using 5% confidence intervals of the false discovery rate (FDR < 0.05). Significant changes in brain regions are visualized using Brain Net Viewer.

### 2.6. Regional Connectivity

Frontoparietal connectivity patterns were analyzed across ten regions of interest (ROIs): right and left frontal, right and left parietal, right and left temporal, right and left occipital, upper midline, and lower midline. The connectivity between these regions was assessed for each condition and frequency band of interest by averaging the corresponding phase lag index (PLI) values. Specifically, for any two regions, i and j, the connectivity was determined by averaging the PLI values of all electrode pairs x, y where x belonged to region i and y belonged to region j. The electrodes assigned to each ROI were as follows: left frontal (FP1, AF3, F3, F7), left temporal (T7, FC5, C3), left partial (CP5, P7, P3), right frontal (FP2, AF4, F4, F8), right temporal (FC6, C4, T8), right partial (CP6, P4, P8), upper midline (Fz, FC1, FC2), lower midline (CP1, CP2, Oz), left occipital (PO3, O1), right occipital (PO4, O2).

## 3. Results

### 3.1. Power Spectral and Connectivity

**Figure 2:**
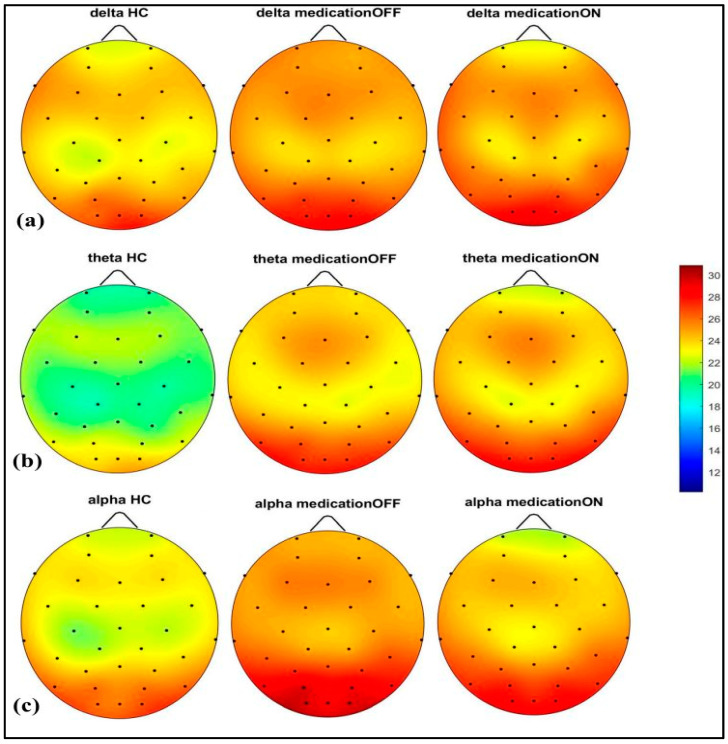
Spectral power representation of resting-state HC, resting-state PD OFF, and resting-state PD ON for delta (**a**), theta (**b**), alpha (**c**).

The spectral power analysis across different frequency bands revealed distinct patterns in the three groups: healthy control, Parkinson’s disease dopaminergic medication OFF, and Parkinson’s disease dopaminergic medication ON. As shown in Figure 2, in the delta (2–4 Hz) and theta (4.1–8 Hz) bands, the Parkinson OFF group exhibited increased power in the prefrontal, left and right centro-parietal, and occipital regions compared to the controls. Following medication (ON state), spectral power showed normalization towards healthy control levels in the prefrontal and centro-parietal areas, evidence of a medication-induced modulation.

**Figure 3:**
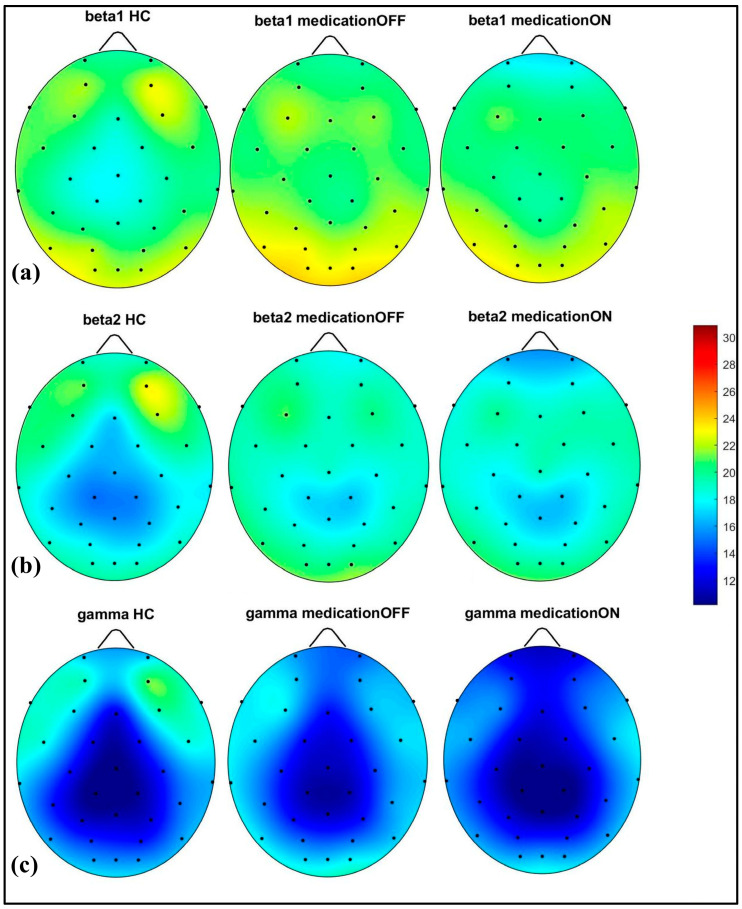
Spectral power representation of resting-state HC, resting-state PD OFF, and resting-state PD ON for beta1 (**a**), beta2 (**b**), gamma (**c**).

In the alpha (8.1–12 Hz) band, there was a notable power value increase in both the left and right occipital regions of the Parkinson OFF group. The observed increase in occipital power, particularly in the alpha band for the Parkinson OFF group, may be attributed to the resting-state condition with eyes open during the EEG recordings. It is well established that cerebral activity, especially in the occipital lobe, is strongly modulated by the eye condition [33]. Specifically, alpha activity is more pronounced in the occipital region during the eyes-closed state and tends to decrease with eyes open. However, in Parkinson’s disease, there may be a compensatory increase in occipital alpha power during the eyes-open condition. Medication normalized these in the prefrontal, mid-parietal, and occipital regions. As shown in Figure 3, for the beta1 (12.1–20 Hz) and beta2 (20.1–30 Hz) bands, a distinct pattern was identified at the prefrontal and fronto-central–parietal midline in the healthy control group. In contrast, the Parkinson OFF group showed decreased power in the left and right frontal regions, with increased activity in the occipital and central–parietal midline areas. Medication led to normalization towards healthy control levels at the parietal and occipital regions, though a decrease in power was observed in the prefrontal areas, where spectral power decreased below the control group’s levels. Finally, in the gamma band (30.1–47 Hz), a similar trend of power reduction in the frontal regions and increased activity in the occipital area was noted for the Parkinson OFF group. Medication led to the most significant normalization towards healthy control levels in the prefrontal and fronto-central–parietal midline.

### 3.2. Graph Theory

**Figure 4:**
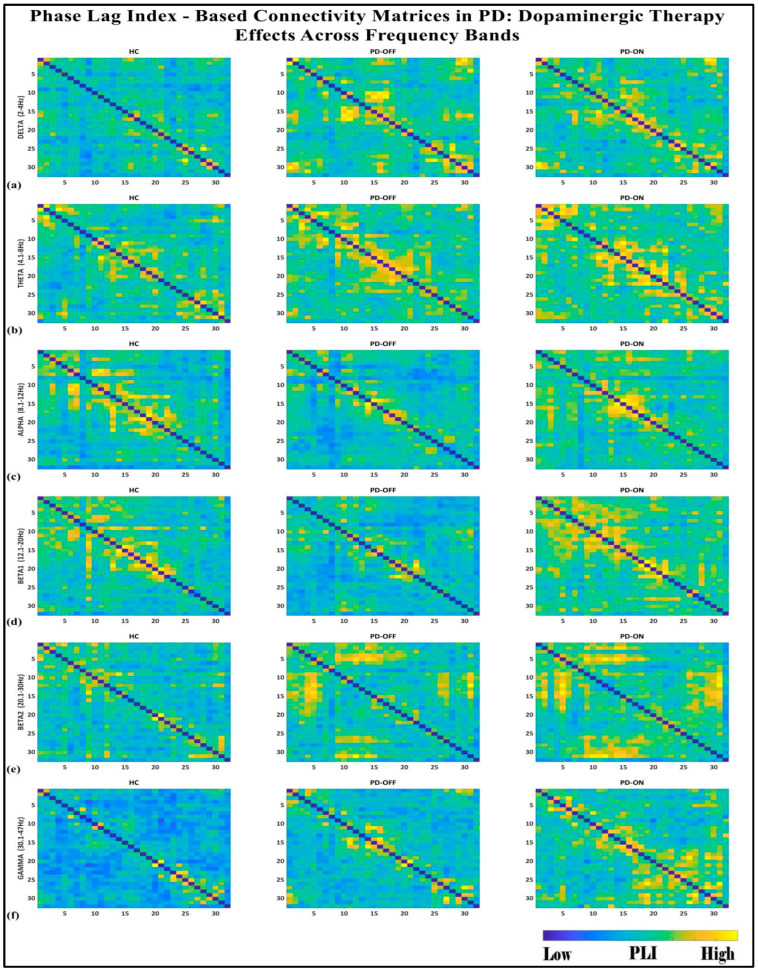
PLI connectivity matrices for healthy control (HC), Parkinson without medication (OFF), and Parkinson with medication (ON) for (**a**) delta, (**b**) theta, (**c**) alpha, (**d**) beta1, (**e**) beta2, (**f**) gamma.

**Figure 5:**
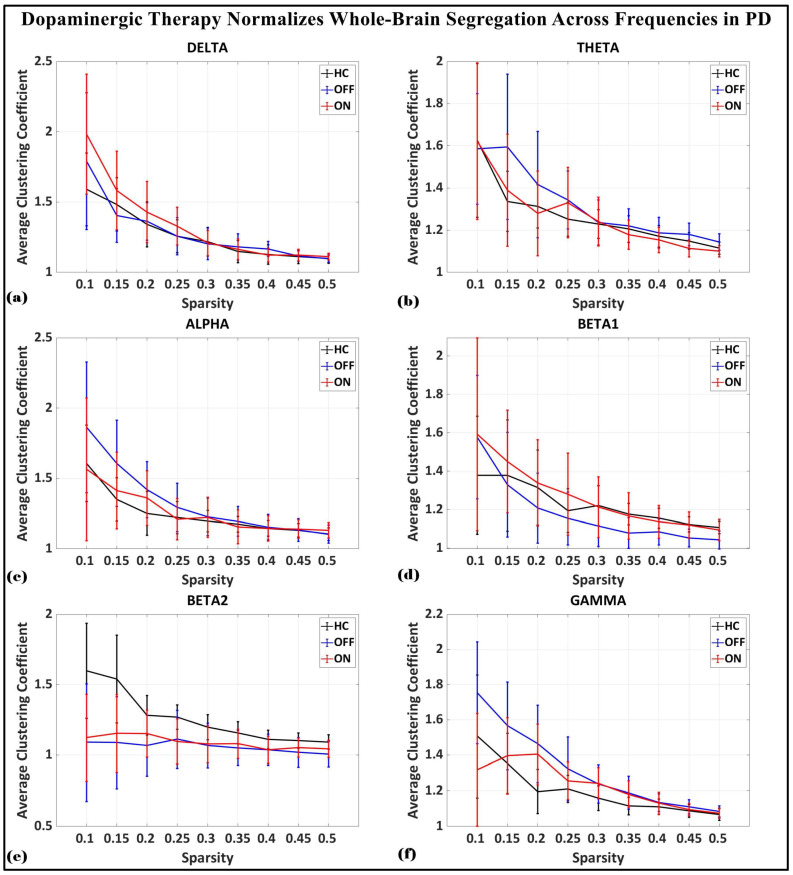
Average clustering coefficient (whole-brain segregation) of brain network topology for healthy control (CTL) (black), Parkinson without medication (OFF) (blue), and Parkinson with medication (ON) (red) for (**a**) delta, (**b**) theta, (**c**) alpha, (**d**) beta1, (**e**) beta2, (**f**) gamma.

**Figure 6:**
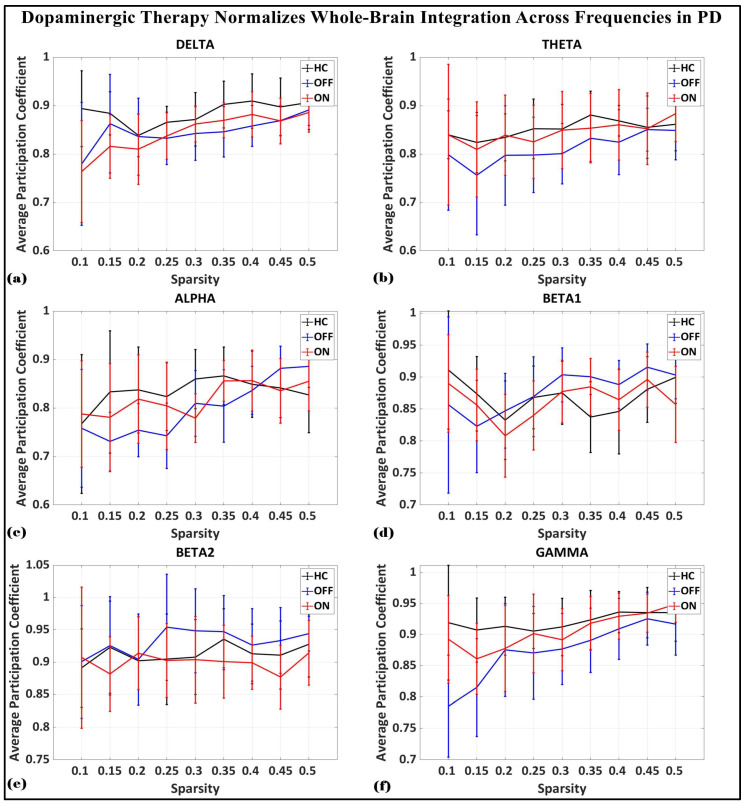
Average participation coefficient (whole-brain integration) of brain network topology for healthy control (CTL) (black), Parkinson without medication (OFF) (blue), and Parkinson with medication (ON) (red) for (**a**) delta, (**b**) theta, (**c**) alpha, (**d**) beta1, (**e**) beta2, (**f**) gamma.

**Figure 7:**
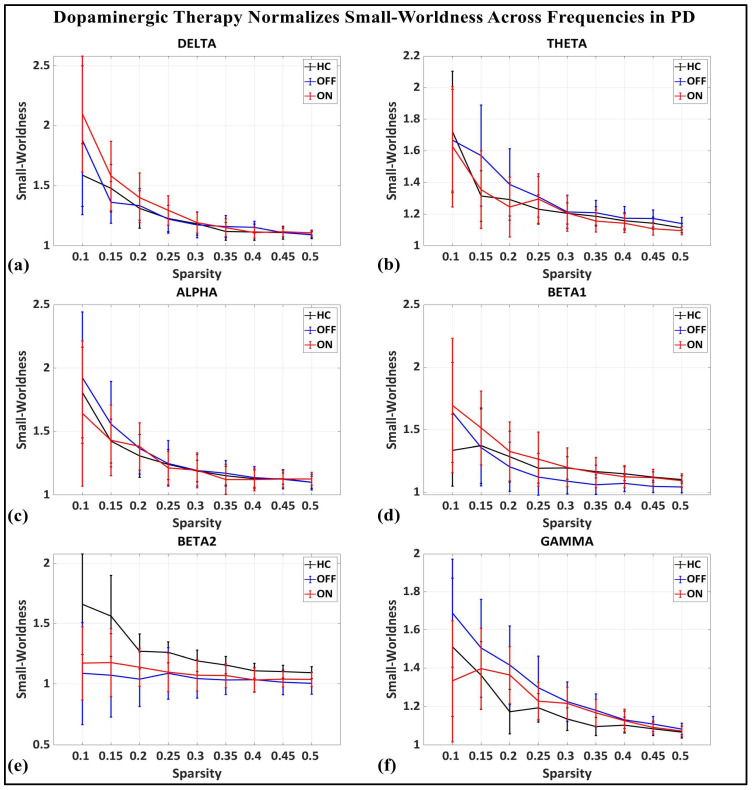
Small-worldness of brain network topology for healthy control (CTL) (black), Parkinson without medication (OFF) (blue), and Parkinson with medication (ON) (red) for (**a**) delta, (**b**) theta, (**c**) alpha, (**d**) beta1, (**e**) beta2, (**f**) gamma.

To investigate the network-level connectivity, we constructed a PLI connectivity matrix based on the inter-relationship between all pairs of electrodes, as shown in Figure 4. The graph theory analysis across the three groups revealed a distinct plot of whole-brain network segregation, integration, and small-worldness. In terms of the average clustering coefficient, the healthy control (HC) and medication ON (ON) groups followed similar trajectories across the sparsity levels, reflecting the normalization of brain connectivity in the Parkinson’s ON group, particularly in the beta band after a sparsity threshold of 0.33, as shown in Figure 5. This suggests that dopaminergic medication restores the clustering coefficient in the brain networks to a level comparable with the healthy controls. However, especially the delta band, the ON and OFF groups showed similarities, reflecting incomplete network restoration under medication. As shown in Figure 6, for the average participation coefficient, a similar normalization value was observed between the HC and ON groups, with the ON group’s participation coefficient aligning closely with the healthy control values, especially after 0.33 sparsity in the beta band. This demonstrates that medication induces a restoration of inter-network communication and functional integration. Finally, the small-worldness analysis revealed a comparable plot where the HC and ON groups showed aligned small-world properties at higher sparsities, as shown in Figure 7. This alignment indicates that dopaminergic medication helps restore the optimal balance between integration and segregation, which is critical for efficient brain function.

### 3.3. Boxplot of Segregation, Integration, Small-Worldness

**Figure 8:**
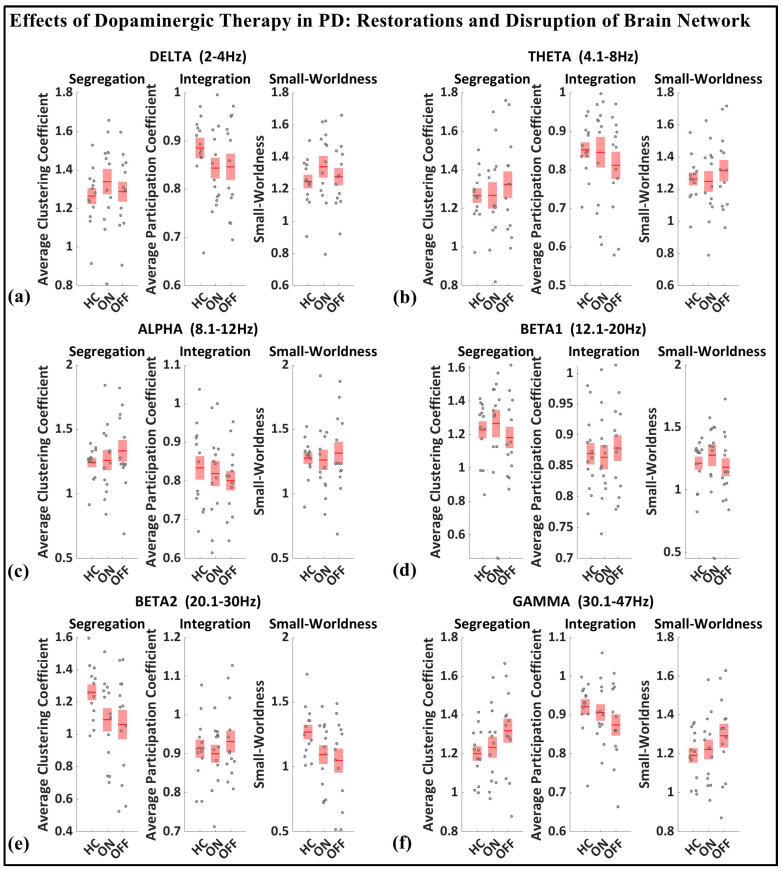
Boxplot of whole-brain segregation, integration, and small-worldness for healthy control (HC), Parkinson with medication (ON), and Parkinson without medication (OFF) for (**a**) delta, (**b**) theta, (**c**) alpha, (**d**) beta1, (**e**) beta2, (**f**) gamma frequency.

As shown in Figure 8, based on the median values presented for the whole-brain segregation (average clustering coefficient), integration (average participation coefficient), and small-worldness properties across six frequency bands (delta, theta, alpha, beta1, beta2, gamma) in the healthy controls, Parkinson’s patients in the medication OFF state, and Parkinson’s patients in the medication ON state, we can observe the following pattern.

In the medication ON group, certain graph theory measures show a normalization effect towards the healthy control state when compared to the medication OFF group. For instance, in the theta and alpha bands, the segregation values of the medication ON group closely approximate those of the healthy controls, indicating a restoration of local connectivity often disrupted in Parkinson’s disease. Similarly, the small-worldness values in the theta, alpha, beta, and gamma bands for the medication ON group revert to levels similar to those of the healthy controls, suggesting that dopamine medication may enhance the balance between global and local brain network efficiency, resembling a more physiologically typical network configuration. These patterns imply that dopaminergic therapy plays a significant role in modulating brain connectivity dynamics, promoting network efficiency that aligns more closely with the healthy brain, particularly in frequency bands that support sensorimotor functions.

In contrast to the normalization effects observed in other frequency bands following dopaminergic therapy administration, the delta band presents a notable deviation. Specifically, segregation and small-worldness in the delta band increase abnormally in the medication ON state compared to the OFF state. This could be indicative of increased local connectivity and modularity, potentially reflecting compensatory mechanisms linked to non-motor symptoms such as cognitive decline and sleep disorders, both of which are associated with elevated delta connectivity segregation in Parkinson’s disease (PD). The increased small-worldness may represent heightened local clustering and reduced global efficiency, a configuration commonly associated with early-stage cognitive impairment. Furthermore, these changes could point to the disruptive influence of dopaminergic therapy on sleep regulation, as PD patients often experience sleep-related issues such as insomnia and excessive daytime sleepiness following medication. As such, the abnormal response of the delta band reflects a combination of disease-related network dysfunction and medication-induced alterations in brain dynamics.

Table 1, Table 2 and Table 3 reveal nodal-level frequency-specific connectivity changes across different brain regions in Parkinson’s disease (PD) patients in the ON and OFF medication states compared to the healthy controls (CTL). As shown in Table 1, in the delta band, OFF vs. CTL showed increased prefrontal connectivity at Fp1 (*p* = 0.003) and decreased connectivity at Pz (*p* = 0.0166). ON vs. OFF showed increased prefrontal connectivity at Fp2 (*p* = 0.016), as shown in Table 3. As per the data presented in Table 1 and Table 2, in the alpha band, FC2 (*p* < 0.001) showed increased connectivity in OFF vs. CTL and ON vs. CTL. Similarly, the beta1 band exhibited increased connectivity at FC6 (*p* = 0.01033) in ON vs. CTL. In contrast, the beta2 band showed decreased connectivity across multiple regions (P7, PO3, F4) in both OFF vs. CTL and ON vs. CTL. ON vs. CTL showed decreased connectivity at FC2 (*p* < 0.00019). However, in the gamma band, connectivity at F3 and C4 was increased in OFF vs. CTL and connectivity at P8 (*p* < 0.013) increased in ON vs. CTL.

**Figure 9:**
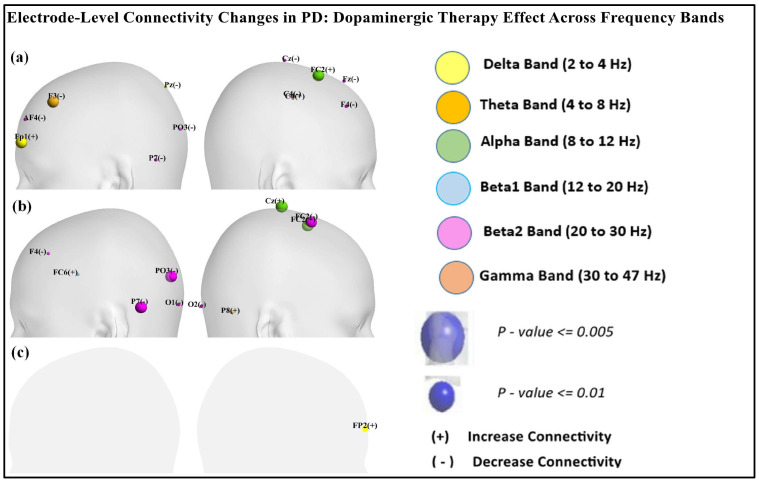
Scalp visualization of the brain regions (electrodes) showed significant difference in the integrated nodal clustering coefficient values (γ) in (**a**) resting-state PD OFF compared to CTL, (**b**) resting-state PD ON compared to CTL, (**c**) resting-state PD ON compared to PD OFF. Nodal color indicates different frequency bands.

Figure 9 shows the scalp visualization of brain regions (electrodes) with significant differences in the integrated nodal clustering coefficient values. Panel (a) highlights differences between resting-state PD OFF and CTL, panel (b) compares resting-state PD ON to CTL, and panel (c) contrasts resting-state PD ON with PD OFF. Nodal color in the figure indicates different frequency bands.

**Figure 10:**
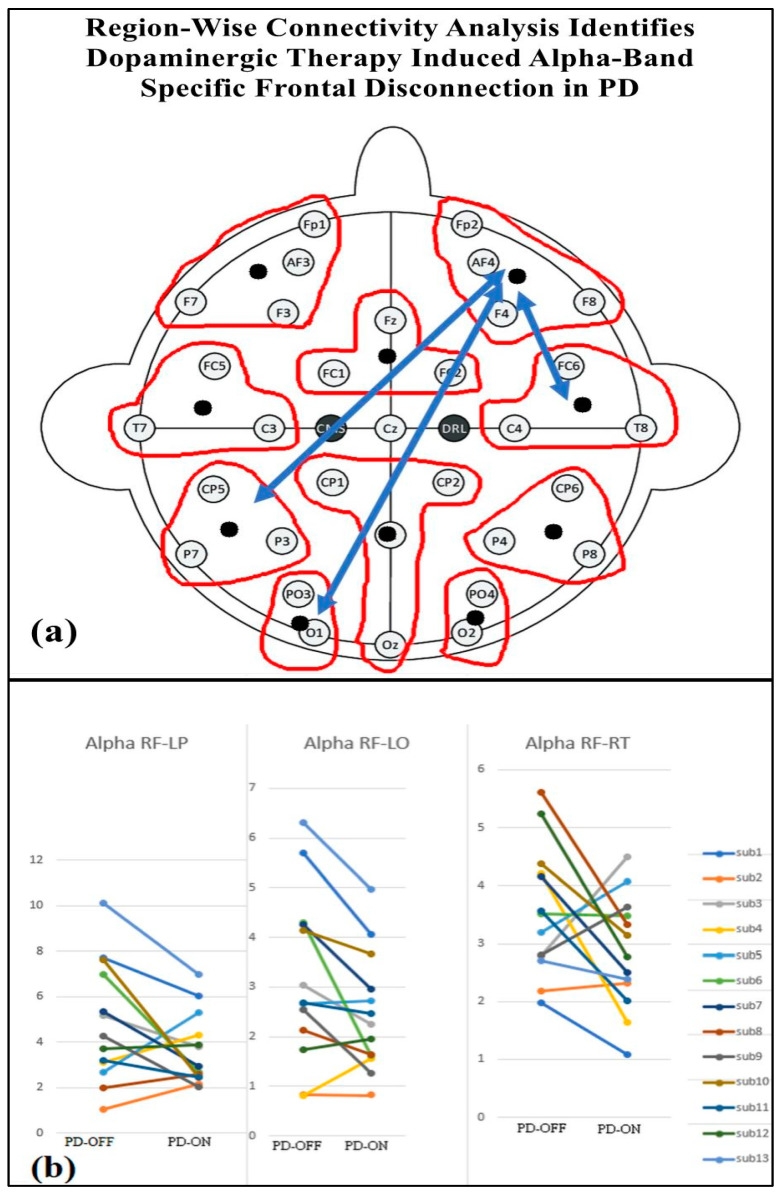
(**a**) Regional brain connectivity alteration after medication ON. (**b**) Decreased right frontal (RF) connectivity with left parietal (LP), left occipital (LO), and right temporal regions (RT) after medication ON compared with medication OFF.

Table 4 presents the regional-wise brain connectivity analysis in the alpha band, highlighting connectivity changes between the right frontal region and other brain regions in the PD-OFF and PD-ON conditions. The results indicate a significant decrease in connectivity with the left parietal, left occipital, and right temporal regions after dopaminergic medication. This reduction in long-range connectivity is further illustrated in Figure 10, which provides the EEG topographical representation of these changes. The findings collectively suggest altered network organization and disrupted communication between frontal and posterior regions, emphasizing the impact of dopaminergic medication on regional brain connectivity in Parkinson’s disease.

## 4. Discussion

Current treatment options for Parkinson’s disease (PD) often provide limited effectiveness over prolonged periods, with outcomes that vary significantly among patients. While dopamine-targeted medications have shown some promise, particularly for motor symptoms, there is still much to understand about their mechanisms and the broader neural impact [1]. This study addresses these gaps by examining the effects of dopaminergic therapies on brain neural connectivity to determine their influence on PD symptoms and network connectivity. A key objective was to analyze how these treatments impact brain connectivity and frequency bands. To achieve these insights, we used resting-state EEG data that allow for a detailed analysis of brain connectivity in relation to patients’ responses to dopaminergic treatment [34]. This method enables an exploration of the frequency-specific and network-wide effects of dopamine-based therapies, offering a more nuanced understanding of how these interventions affect brain connectivity in PD. In clinical practice, the Unified Parkinson’s Disease Rating Scale (UPDRS) remains the gold standard for assessing motor function, offering a well-established framework for clinical evaluations. However, neurophysiological techniques, like resting-state EEG, provide a more comprehensive view by identifying additional neurobiological markers of PD beyond motor abilities. Nonetheless, variability in study results highlights the need to establish a unified model of PD to improve diagnostic accuracy and to guide more targeted therapeutic approaches [35].

One factor potentially contributing to this variability is the extensive loss of nerve connections and neurons commonly observed in PD. This degeneration leads to reduced baseline synaptic activity within dopaminergic circuits, emphasizing the importance of restoring function in these regions to facilitate motor and cognitive recovery [25]. Using resting-state EEG, we assessed the effects of dopaminergic therapies on power spectral and connectivity analysis, providing insights into how dopaminergic treatments may normalize or alter brain networks across various frequency bands. This investigation examined the recovery mechanisms in PD, identifying specific biomarkers that may help predict positive responses to therapy. Such biomarkers are valuable for enabling tailored treatment plans, where therapies can be adjusted based on individual neurophysiological characteristics [8].

Dopaminergic treatment led to normalization effects across multiple frequency bands, with enhanced brain connectivity aligning more closely with those observed in healthy individuals [36]. Our results extend the findings of Vecchio et al. (2021), who also reported small-worldness alterations in PD using EEG graph theory, particularly in the theta and alpha bands [37]. This corroborates the work of Li et al. (2021), who found a similar normalization effect of beta1 and beta2 band connectivity following DBS in PD patients [38]. This observation supports the idea that dopaminergic therapies may facilitate network restoration, particularly within motor-related frequency bands. By characterizing the networks influenced by dopamine-based treatment, we identified consistent features in different frequencies and connectivity, which provides valuable insights into the mechanisms of neuromodulation. The impact of this therapy extends beyond motor symptom relief, affecting various network dynamics in both beneficial and maladaptive ways.

Dopaminergic therapy is particularly effective in restoring functionality in the high-frequency beta and gamma bands, which are closely associated with motor control and sensory–motor integration [14,39]. From a neurophysiological perspective, these changes can be interpreted through graph theory measures. The observed increase in the participation coefficient in the beta and gamma bands suggests enhanced global network integration, enabling more efficient communication between distant motor and sensory areas. Additionally, reduced path lengths in these frequency bands indicate a more optimized and efficient flow of motor-related information, leading to improved motor control and execution. Previous studies underscore that increased participation coefficients and reduced path lengths in beta and gamma frequencies are associated with optimized sensory–motor processing, underscoring the efficacy of dopaminergic therapy in enhancing motor performance [14,39].

However, while dopaminergic therapy supports motor function improvement, its impact on non-motor symptoms is more complex, with abnormalities observed in the low-frequency delta band. In this study, the increased clustering coefficient in the delta band suggests excessive local connectivity, leading to abnormal segregation of functional modules. This aberrant network configuration may contribute to the persistence of depressive symptoms in PD patients despite dopamine therapy. This paradoxical increase in delta band segregation during dopaminergic therapy points to dysfunction in the brain [40,41]. In this study, the Beck Depression Inventory (BDI) scores were higher in both the OFF (9.44 ± 5.07) and ON (7.76 ± 5.05) medication states compared to the controls (3.27 ± 3.20), showing significant differences: t(27) = 3.05, *p* < 0.01 (OFF vs. controls) and t(30) = 4.25, *p* < 0.005 (ON vs. controls).

Studies by Zhou et al. 2023 and Whalen 2021 corroborate the above findings, demonstrating that delta abnormalities are strongly associated with depressive symptoms and emotional dysregulation [40,42]. The delta band emerges as a potential biomarker for depression in PD, with the capacity to guide personalized treatment approaches that address these symptoms alongside motor improvements.

Our findings revealed a significant decrease in alpha band connectivity between the right frontal region with several other brain regions (left parietal, left occipital, and right temporal) in PD patients in the OFF state compared to the ON state. A neurophysiological interpretation suggests that this reduction in connectivity reflects impaired network integration. The frontal cortex, which plays a key role in attention and executive function, may fail to effectively coordinate with posterior brain regions, leading to cognitive deficits [43]. This reduction in frontal alpha connectivity may be indicative of cognitive impairment, as alpha oscillations are known to play a critical role in attention, working memory, and executive functioning [44,45]. In Parkinson’s disease, cognitive deficits are often linked to the dysregulation of frontoparietal and fronto-occipital networks, which are essential for integrating sensory information and maintaining cognitive control [46,47,48].

Decreased frontal alpha connectivity in Parkinson’s disease is linked to impairment [49]. Frontal dysfunction also contributes to levodopa-induced dyskinesia, suggesting dopamine therapy alters network dynamics beyond motor symptoms [50]. These changes may also underlie impulse control disorders in treated patients [51].

The results of this study underscore the importance of modern treatment approaches, as standard dopaminergic therapy may inadequately cure the full spectrum of PD symptoms. For instance, while dopaminergic therapy restores motor network function through enhanced beta and gamma connectivity, the persistent delta band abnormalities indicate that dopaminergic treatment alone may not suffice for PD patients. The observed alterations in network topology, including increased clustering in the delta band and reduced global integration in the alpha band, highlight the complexity of dopaminergic effects on brain function. This limitation calls for the integration of complementary therapies, such as cognitive–behavioral therapy or non-invasive brain stimulation, which have shown promise in addressing non-motor symptoms like depressive states and cognitive decline. The non-pharmacological treatments may be effective in mitigating depressive symptoms and improving cognitive functions when used alongside dopaminergic therapy [52,53]. By addressing delta band activity through a multimodal approach, it may be possible to manage mood disturbances and emotional dysregulation more effectively.

Personalized medicine holds promise for PD, especially in the light of findings that demonstrate differential network responses to dopaminergic therapy. Given the persistence of delta band abnormalities, individualized approaches that monitor delta oscillations as real-time biomarkers could help tailor treatment strategies, optimizing dopaminergic dosage while supplementing with complementary therapies. The use of biomarkers like delta oscillations for the early detection of mood disturbances offers a framework for designing targeted interventions that improve patients’ outcomes. Graph-theoretical insights into PD suggest that future therapies should aim not only to restore motor-related network integration but also to rebalance segregation and integration in non-motor-related networks. The delta band activity could be incorporated into treatment monitoring, allowing clinicians to adapt therapeutic approaches to individual symptom profiles [40,54]. Such personalized interventions would represent a major advancement in PD management, aligning treatment with the unique network dynamics of each patient to optimize outcomes.

Current Source Density (CSD) transformation enhances spatial localization by reducing volume conduction effects and emphasizing local neural activity while minimizing global interference. This approach is particularly beneficial for improving the accuracy of functional connectivity analysis in graph theory by reducing spurious long-range connections. However, CSD also has limitations, including potential distortions in low-frequency components (delta and theta bands) and edge effects at electrode borders. Additionally, its effectiveness depends on electrode density and spatial interpolation accuracy. While CSD could refine network topology measurements in Parkinson’s disease, its impact on graph-theoretical metrics warrants further investigation in future studies.

### Limitations and Future Study

This study has several limitations that must be acknowledged. Firstly, the use of a low-density EEG setup restricted our ability to examine the finer spatial patterns of brain connectivity. Additionally, while a higher electrode density can enhance spatial resolution, it may also increase the risk of volume conduction effects, where signals from a single neural source are detected by multiple nearby electrodes, potentially inflating connectivity estimates. Future studies could employ source localization or other signal-processing techniques to minimize such confounds. The variability in medication timing among patients may have affected the results. The small, heterogeneous sample size contributed to variability in outcomes, particularly in distinguishing between true treatment effects and individual differences in disease progression. Additionally, the withdrawal period for antiparkinsonian drugs may not fully capture long-term dopaminergic responses. The absence of a control group receiving levodopa complicates our ability to isolate its specific effects, particularly in disentangling medication-related changes from disease-related connectivities. Lastly, variations in medication dosage among participants also pose a potential confounding factor, emphasizing the need for dose–response analyses in future work. In future studies, we propose to further investigate the characteristics of both responder and non-responder groups to uncover baseline factors that may predict responsiveness to dopaminergic therapy. Longitudinal designs with high-density EEG, combined with multimodal imaging (e.g., fMRI or PET), could clarify the dynamics of network reorganization under dopaminergic modulation. Additionally, we plan to explore the phenomenon of false negative non-responders, particularly in patients who display increased metabolism and connectivity despite not showing improvements in motor function. This could help refine our understanding of treatment outcomes and optimize therapeutic strategies.

## 5. Conclusions

This study provides the impact of dopaminergic therapy in PD, demonstrating that dopaminergic therapy normalizes graph-theoretic metrics in most frequency bands but exacerbates delta band connectivity continuously from HC to PD-OFF to PD-ON. The normalization observed in the beta and gamma bands supports improved motor function and sensory–motor integration, while persistent delta band dysfunction relates with depressive symptoms or sleep disturbances. Additionally, reduced frontal alpha connectivity in regional-wise analysis may contribute to cognitive impairment and levodopa-induced dyskinesia, highlighting its relevance in Parkinson’s disease progression These findings highlight the need for personalized treatment strategies that address overall patient outcomes.

Future research should focus on developing integrative therapeutic approaches, combining pharmacological treatments with cognitive and behavioral therapies to mitigate the adverse effects of dopaminergic therapy on non-motor symptoms. Additionally, the delta band dynamics observed in this study could serve as a biomarker for mood disturbances and treatment response, providing a framework for tailored interventions that optimize both motor and non-motor outcomes. Overall, this study underscores the importance of understanding the complex interaction between network segregation and integration in PD, paving the way for more effective and comprehensive treatment strategies.

## Figures and Tables

**Table 1:** Significant brain regions computed from nodal brain segregation (clustering coefficients) for OFF vs. CTL.

Freq Band	Electrodes	OFF < CTL (Effect Size)	OFF < CTL (Cohen’s d)	OFF < CTL (*p*-Value)	95% Confident Interval	Connectivity
DELTA	Fp1	0.4993	1.1528	0.003	[−0.342, −0.068]	increase
	Pz	−0.4047	−0.8853	0.0166	[0.024, 0.338]	decrease
ALPHA	FC2	0.5461	1.3039	0.00142	[−0.400, −0.103]	increase
BETA2	P7	−0.3922	−0.8527	0.01988	[0.016, 0.311]	decrease
	PO3	−0.4445	−0.9926	0.00919	[0.040, 0.315]	decrease
	C4	−0.4334	−0.96194	0.01094	[0.055, 0.493]	decrease
	F4	−0.4689	−1.0617	0.00617	[0.072, 0.447]	decrease
	AF4	−0.4565	−1.0264	0.00757	[0.060, 0.420]	decrease
	Fz	−0.4215	−0.9298	0.01308	[0.031, 0.324]	decrease
	Cz	−0.4073	−0.8919	0.0161	[0.045, 0.609]	decrease
GAMMA	F3	−0.4752	−1.0801	0.00554	[−0.496, −0.083]	decrease
	C4	0.3968	0.8647	0.0187	[−0.304, −0.018]	increase

**Table 2:** Significant brain regions computed from nodal brain segregation (clustering coefficients) for ON vs. CTL.

Freq Band	Electrodes	ON < CTL (Effect Size)	ON < CTL (Cohen’s d)	ON < CTL (*p*-Value)	95% Confident Interval	Connectivity
ALPHA	FC2	0.5568	1.34098	0.00113	[−0.339, −0.092]	increase
	Cz	0.4754	1.0809	0.0055	[−0.595, −0.100]	increase
BETA1	FC6	0.4371	0.97218	0.01033	[−0.390, −0.046]	increase
BETA2	P7	−0.4865	−1.1138	0.0045	[0.069, 0.376]	decrease
	PO3	−0.474	−1.0766	0.00566	[0.061, 0.365]	decrease
	O1	−0.4251	−0.9395	0.0124	[0.036, 0.357]	decrease
	O2	−0.4301	−0.9529	0.0115	[0.049, 0.460]	decrease
	FC2	−0.6285	−1.6161	0.00019	[0.168, 0.474]	decrease
	F4	−0.4186	−0.922	0.01367	[0.034, 0.370]	decrease
GAMMA	p8	0.4201	0.9259	0.013	[−0.365, −0.034]	increase

**Table 3:** Significant brain regions computed from nodal brain segregation (clustering coefficients) for ON vs. OFF.

Freq Band	Electrodes	ON < OFF (Effect Size)	ON < OFF (Cohen’s d)	ON < OFF (*p*-Value)	95% Confident Interval	Connectivity
DELTA	Fp2	0.4044	0.8844	0.016	[0.020, 0.290]	increase

**Table 4:** Connectivity of the right frontal regions with other brain regions in the alpha band for PD-OFF and PD-ON groups. Statistical significance is reported for all between pairwise comparisons, with FDR-corrected *p*-values (*p* < 0.05).

	Regions	PD-OFF	PD-ON	*p*-Value	Connectivity
ALPHA	Right frontal to left partial	4.84 ± 2.63	3.65 ± 1.58	0.0432	decrease
	Right frontal to left occipital	3.16 ± 1.70	2.45 ± 1.19	0.008	decrease
	Right frontal to right temporal	3.56 ± 1.11	2.83 ± 0.97	0.041	decrease

## Data Availability

The original data presented in the study are openly available in the Openneuro database at https://openneuro.org/datasets/ds002778/versions/1.0.0 (accessed on 24 July 2024), accession number ds002778.
